# Highly brominated anthracenes as precursors for the convenient synthesis of 2,9,10-trisubstituted anthracene derivatives

**DOI:** 10.3762/bjoc.4.50

**Published:** 2008-12-10

**Authors:** Osman Cakmak, Leyla Aydogan, Kiymet Berkil, Ilhami Gulcin, Orhan Buyukgungor

**Affiliations:** 1 Gaziosmanpasa University, Faculty of Art and Science, Department of Chemistry, TR-60240, Tokat, Turkey; 2 Ataturk University, Faculty of Art and Science, Department of Chemistry, TR-25240, Erzurum, Turkey; 3 Ondokuzmayis University, Faculty of Art and Science, Department of Physics, TR-55060, Samsun, Turkey (author to whom inquries concerning the X-ray structure should be directed)

**Keywords:** anthracene derivative, bromination, bromoanthracene, cyanoanthracene, methoxyanthracene

## Abstract

When 9,10-dibromoanthracene was treated with bromine in CCl_4_ without a catalyst, 1,2,3,4,9,10-hexabromo-1,2,3,4-tetrahydroanthracene (**3**) was obtained in 95% yield in the absence of other stereoisomers or rearomatization products. We investigated the base-induced elimination reaction of hexabromide **3** under various conditions. Pyridine-induced elimination of hexabromide **3** afforded 2,9,10-tribromoanthracene (**12**) in 75% yield, and tribromide **12** was transformed to trimethoxy compound **13** and trinitrile **14** by copper-assisted nucleophilic substitution reactions.

## Introduction

Anthracene derivatives have been extensively investigated in many fields, e.g., material chemistry [1–4], thermochromic or photochromic fields [5], and organic light-emitting devices [6–14]. Moreover, anthracenes have been used in optical, electronic, and magnetic switches, and combined with polymers, films, and crystals [15–16]. In biological systems, anthracene skeletal compounds are also useful for probing DNA cleavage [17].

Bromoanthracenes have become increasingly important in the synthesis of anthracene derivatives [18]. For example, new anthracene derivatives used as light emitting material in a non-doped organic light-emitting diode (OLED) were synthesized from the corresponding bromo derivatives by substitution [19–23].

Recently we succeeded in the bromination of anthracene to give hexabromides **3** and **4** which were used in the selective and specific preparation of anthracene oxides and anthracene derivatives difficult to prepare by other routes [24]. On the other hand, in a previous study, we isolated stereoisomeric hexabromide **3** from a complex reaction mixture of photobromination of 9,10-dibromoanthracene (**2**) in which the structure of **3** was established by X-ray analysis [25]. We now wish to report on the successful synthesis of hexabromide **7** as the sole product of the reaction in nearly quantitative yield and its base-induced elimination leading to 2,9,10-tribromoanthracene (**12**).

## Results and Discussion

**Scheme 1:**
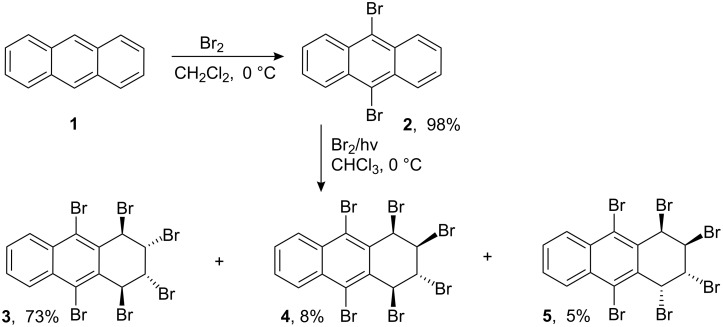
Photobromination of 9,10-dibromoanthracene.

The starting dibromide **2** was prepared from anthracene according to our previously described method [24]. First, bromination of dibromide **2** was examined at 0 °C in CHCl_3_ by irradiation with a sun lamp (Table 1, entry 1). The reaction resulted in the formation of three diastereomers: **3**, **4**, and **5**. After chromatography and fractional crystallization, hexabromides **3**, **4**, and **5** were isolated in yields of 73%, 8%, and 5%, respectively (Scheme 1). The structure of hexabromide **4** was assigned by comparing with an authentic sample, which is the main product in the polar bromination of dibromide **2** (Scheme 1) [24].

**Table 1:** Bromination of 9,10-dibromoanthracene (**2**) with molecular bromine (2.5 equiv).

Entry	Reaction conditions	Time	Conversion	Yield (**3**)	Ratio **3**:**4**:**5**^a^

1	CHCl_3_/hν, 0 °C	4 h	99%	73%	80:13:6
2	CH_2_Cl_2_/hν, 0 °C	2 h	98%	85%	88:5:3
3	CH_2_Cl_2_, 25 °C	5 d	99%	86%	91:4:4
4	Benzene/SiO_2_, 25 °C	4 d	90%	74%	80:6:4
5	CCl_4_/hν, 25 °C	1 h	100%	95%	98:1:1

^a^Ratio of the products was established by ^1^H NMR spectroscopy. A 150 W projector lamp was used in the photolytic reactions (entries 1, 2, and 5).

**Figure 1:**
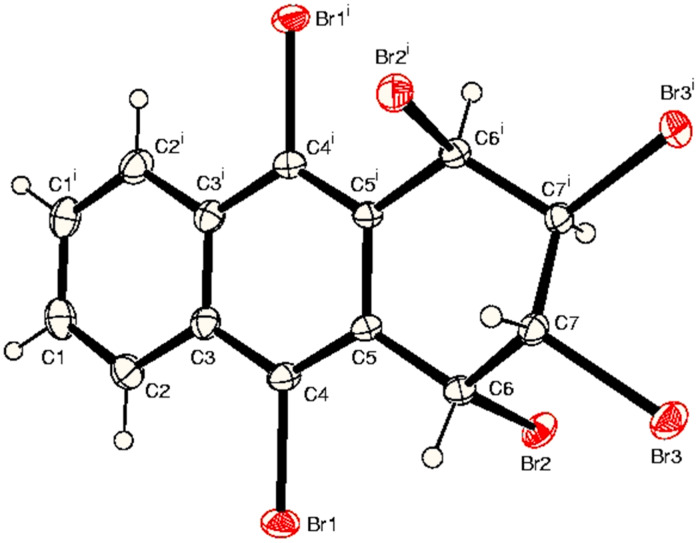
ORTEP view of hexabromide 5.

The structural assignments of hexabromides **3** and **5** were based on correct elemental analysis, parent ion peak in the MS, and simple characteristic ^1^H NMR spectra that exhibited two AA′BB′ systems. The seven lines in the ^13^C NMR spectra also agree with the symmetrical hexabromides **3** and **5**. Four symmetrical stereoisomers can be formed in the reaction. However, on the basis of NMR data alone we are not able to distinguish between the four possible hexabromides. X-ray analysis of hexabromide **3** showed a *trans,cis,trans* relationship of bromine atoms [25]. We also have the crystallographic analysis of hexabromide **5**, which is in accord with *cis,trans,cis* configuration of the bromine atoms in **5**, which has the *aeea* conformation (Figure 1) [26].

**Scheme 2:**
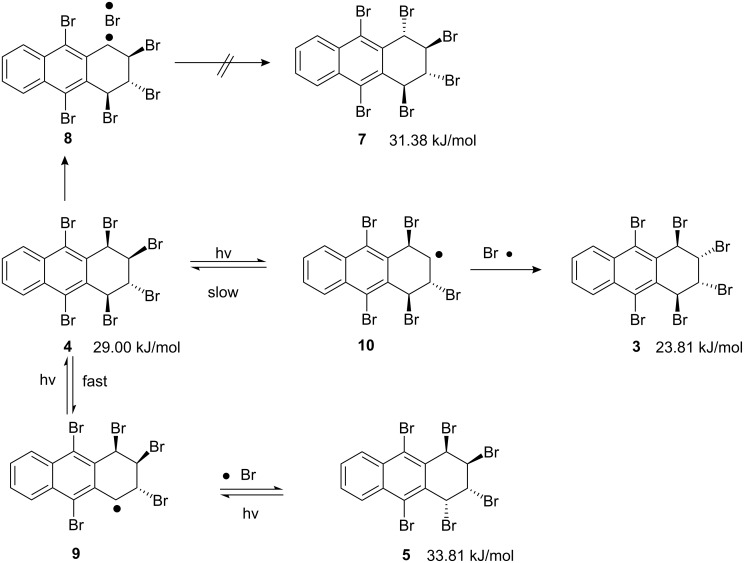
Configuration isomerization mechanism of the hexabromides.

Another photolytic process using methylene chloride (Table 1, entry 2 and entry 3) and silica gel supported bromination (Table 1, entry 4) afforded mainly hexabromide **3**, in addition to minor amounts of **4** and **5**. However, photobromination of dibromide **2** in CCl_4_ is an effective and convenient method to prepare hexabromide **3**. Most of the product precipitated during the reaction. After the reaction, a rapid and simple recrystallization gave the pure hexabromide **3** in 95% yield. As hexabromide **4** is quite sensitive to daylight and temperature, aromatization and epimerization occur to give a product mixture, and compound **3** is more stable. Its strain energy [27] (SE) is the lowest (SE = 23.81 kJ/mol total strain energy, Scheme 2) among the possible stereoisomers, which may explain why compound **3** is selectively formed.

When the pure hexabromide **4** was subjected to daylight at room temperature in solvent (CDCl_3_), an equilibrium was established with the other hexabromides **3** and **5** (Table 2). A similar distribution was observed in the case of pure **5**. In the case of the direct irradiation of **4** with a projector lamp (150 W), equilibrium was established in a short time (15 min). The configurational isomerization stopped in the dark, which strongly supports a radical mechanism for interconversion of the bromides. We assume that the visible light initiates formation of radicals at C-1 and C-3 positions (Scheme 2), while recombination of the radicals (**9** and **10**) causes configuration isomerization to give hexabromides **3** and **5**.

**Table 2:** Irradiation of hexabromide **4**.

Entry	Conditions	Ratio^a^			
		**4**	**5**	**3**	**2**

1	daylight, 1 d	51	41	5	3
2	daylight, 3 d	43	42	6	9
3	daylight, 22 d	43	41	7	9
4	projector lamp, 15 min	41	41	9	9
5	projector lamp, 1 h	33	32	25	10

^a^Ratio of the products was established by ^1^H NMR spectroscopy. The studies were made in an NMR tube in CDCl_3_. A 150 W projector lamp was used in the photolytic reactions.

Isomerization of compound **4** may lead to **3** and **5**. In order to evaluate the relative stabilities of the diastereomeric hexabromides **3, 4,** and **5,** we carried out molecular mechanistic calculations with MM2 relative steric energies [27]. These calculations demonstrate that hexabromide **3** is more stable than the other three (**4**, **5**, and **7**). The fact that hexabromide **5** is the major product instead of the thermodynamically more stable product (hexabromide **3**) may be explained by hexabromide **5** forming via a more stable benzylic radical intermediate (**9**, Scheme 2).

**Scheme 3:**
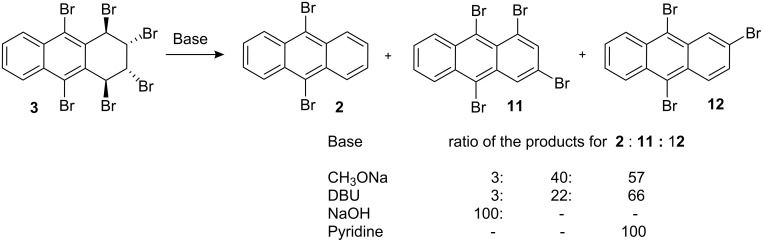
Dehydrobromination of hexabromide **3** with various bases. Relative percentages were calculated by integration of the 1H NMR signals of the compounds.

**Scheme 4:**
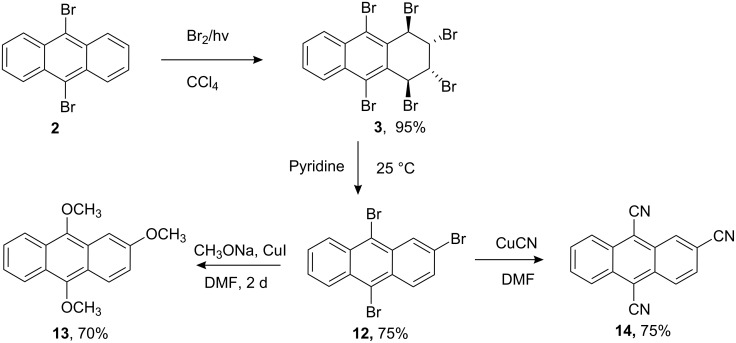
Preparation of trisubstituted anthracene derivatives.

Hexabromide **3** was subjected to aromatization with various bases. Treatment of hexabromide **3** with sodium methoxide or DBU led to a mixture of tetrabromide **11** [24], tribromide **12**, and dibromide **2**, while NaOH produced dibromide **2** (Scheme 3). Lastly, the reaction with pyridine efficiently and selectively afforded 2,9,10-tribromoanthracene (**12**) in high yield (75%, Scheme 4).

Preparation of 2,9,10-tribromoanthracene (**12**) was previously tedious and unsatisfactory [28–30]. Our results show that the bromination and elimination conditions are crucial for selective preparation in high yield. Having obtained tribromide **12** in a high yield, we investigated its value as a precursor of other useful compounds. For this purpose, tribromide **12** was treated with sodium methoxide in the presence of CuI in DMF at ca. 100 °C. The expected trimethoxy compound **13** was isolated as the sole product in 70% yield. The structure of **13** was proved by its ^1^H NMR spectrum. Compound **13** gave a mass spectrum (M^+^) at 268 corresponding to the molecular formula C_17_H_16_O_3_.

Methoxyanthracenes have great synthetic importance in several ways, i.e. in the synthesis of other substituted anthracene derivatives, which can be easily converted to hydroxyanthracenes, and also in the synthesis of anthraquinones. In addition, there are many naturally occurring methoxyanthracene derivatives [31–32].

Copper-assisted nucleophilic substitution of tribromide **12** by cyanide ions easily afforded a cyano derivative of anthracene (**14**) as the sole product (Scheme 4). The IR spectrum exhibited nitrile bands and the correct elemental analysis substantiates the structure of **14** as a trinitrile. The molecular ion at *m/z* 253.0 (base peak, [M^+^]) accompanied with fragment peaks suggest that the structure is a trinitrile. The downfield shift in the proton NMR and seventeen lines in ^13^C NMR are in accord with a trinitrile structure.

## Conclusion

Specific and selective bromination is important in both intermediate and product synthesis. Aromatic bromination generally requires the use of a catalyst and often gives a mixture of products [33]. Therefore, the synthesis of polybromo-substituted anthracenes is restricted because reactivity towards bromine is reduced as bromination proceeds [34]. However, our study achieved efficient bromination without a catalyst under mild conditions.

We have described an efficient and convenient synthesis of the hexabromide **3** and the tribromide **12**, which was produced via base-mediated elimination of **3**. These two compounds are often the starting points for the polyfunctionalization to other anthracene derivatives and as such our methodology offers a new way to prepare a variety of anthracene derivatives in an efficient manner. We demonstrated that the bromination conditions of 9,10-dibromoanthracene dramatically affect the nature of the stereoisomeric hexabromide product and ratio. The studies also revealed that aromatization of hexabromide **3** depends strongly on the choice of base.

## Experimental

### General

Thin layer chromatography was carried out on Merck silica F_254_ 0.255 mm plates and spots were visualized with UV fluorescence at 254 nm. Classic column chromatography was performed using Merck 60 (70-230 Mesh) silica gel. Melting points were determined on a Thomas-Hoover capillary melting point apparatus. Solvents were concentrated at reduced pressure. IR spectra were recorded on a Perkin Elmer 980 instrument. Mass spectra were recorded on a VG Zab Spec GC-MS spectrometer under electron-impact (EI) and chemical ionization conditions. NMR spectra were recorded on a Bruker spectrometer at 400 MHz for ^1^H and at 100 MHz for ^13^C NMR.

### 1. Bromination of 9,10-dibromoanthracene (**2**)

**Entry 1.** 9,10-Dibromoanthracene (**2**, 1.0 g, 2.98 mmol) was dissolved in CHCl_3_ (75 mL) and cooled to 0 °C. To the solution was added dropwise bromine (1.2 g, 7.44 mmol) in CHCl_3_ (10 mL) over 5 min. The solution was irradiated (projector lamp, 150 W) with stirring for 4 h. After removal of the solvent and excess bromine *in* *vacuo* below 5 °C, the residue was recrystallized from CHCl_3_ (10 mL) and hexabromide **3** (1.12 g, 57%) was isolated. The mother liquor was chromatographed on silica gel (90 g) eluted with hexane (*R_f_* = 0.69, 0.40, 0.24 for **3**, **4**, and **5** respectively). Hexabromide **3** (300 mg, total yield 1.42 g, 73%), hexabromide **4** (160 mg, 8%), and hexabromide **5** (100 mg, 5%) were isolated and recrystallized from CHCl_3_/hexane. White solids, melting points for **3, 4**, and **5**, respectively, 178 °C (decomp.), 168 °C (decomp.), and 182 °C (decomp.).

***trans,cis,trans*-1,2,3,4,9,10-hexabromo-1,2,3,4-tetrahydroanthracene (3).** ^1^H NMR (400 MHz, CDCl_3_) δ 8.41 A part of AA′BB′ system, 2 H, H5 and H8), 7.73 (B part of AA′BB′ system, 2H, H_6_ and H_7_), 5.94 (A part of AA′BB′ system, 2H, H_1_ and H_4_), 5.37 (B part of AA′BB′ system, 2H, H_2_ and H_3_); ^13^C NMR (100 MHz, CDCl_3_) δ 135.2, 131.6, 130.5, 130.3, 129.8, 56.7, 54.4; MS (APCI) *m/z* 697.30 (M^+^+H+Na+NH_4_), 617.35 (M^+^+H+Na+NH_4_–Br), 515.30 (M^+^+H+NH_4_–2Br), 453.30 (M^+^+H+NH_4_+Na–3Br), 329.10 (M^+^+H+NH_4_–Na+4Br–C_4_H_4_), 312.20, 285.10, 267.1, 227.10, 143.10, 125.10, 100.10; IR (KBr) ν_max_ 3000, 2980, 1500, 1490, 1310, 1230, 1195, 1122, 1110, 1010, 1005, 980, 903. Anal. calcd for C_14_H_8_Br_6_ (655.6): C: 25.65, H: 1.23. Found C, 25.50; H, 1.20.

***cis,trans,cis*-1,2,3,4,9,10-hexabromo-1,2,3,4-tetrahydroanthracene (5).** ^1^H NMR (400 MHz, CDCl_3_) δ 8.41 (A part of AA′BB′ system, 2H, H_5_ and H_8_), 7.68 (B part of AA′BB′ system, 2H, H_6_ and H_7_), 6.26 (A part of AA′BB′ system, 2H, H_1_ and H_4_), 5.16 (B part of AA′BB′ system, 2H, H_2_ and H_4_), ^13^C NMR (100 MHz, CDCl_3_) δ 135.2, 134.2, 131.8, 130.8, 129.0, 61.7, 54.8; MS (CI) *m/z* 658 (M^+^), 575, 496, 418/416/414/412, 339/338/336/334/333, 258/257/256/254, 177/176, 174/168, 150, 122, 111, 99, 98, 88, 87, 86, 74, 63, 50, 39; Anal. calcd for C_14_H_8_Br_6_ (655.6): C, 25.65; H, 1.23. Found: C, 25.53; H, 1.25.

**Entry 2.** The bromination was carried out in a borosilicate glass cylindrical vessel with two necks, in which a tube was immersed (immersion-well type reactor). For irradiation, this tube contained a 150 W projector lamp, surrounded by a glass tube, and cooled by water circulation. Dibromide **2** (1 g, 2.97 mmol) was dissolved in CH_2_Cl_2_ (70 mL) in the reaction apparatus and cooled to 0 °C. To the solution, which was being irradiated with stirring, was added dropwise Br_2_ (1.19 g, 7.44 mmol) in CH_2_Cl_2_ (10 mL) over 30 min. After completion of the reaction (2 h), the solvent was removed and the residue was filtered from a short silica gel column (15 g) eluting with hexane (45 mL). The product ratio was established as 88:5:3:2 by ^1^H NMR for **3**, **4**, **5**, and **2**, respectively. Hexabromide **3** was obtained in 85% yield (1.68 g) after crystallization of the mixture (CHCl_3_, 70 mL, rt, in the dark).

**Entry 3.** To the solution of dibromide **2** (1 g, 2.95 mmol) in CH_2_Cl_2_ (80 mL) was added bromine (1.9 g, 9.83 mmol) in CH_2_Cl_2_ (10 mL) dropwise over 1 h. The mixture was stirred in daylight at room temperature for 5 d. After removal of the solvent, the residue (2.02 g) was filtered through a short silica gel column (15 g) eluting with hexane (40 mL), and the product ratio was established by NMR as 91:4:4:1 for **3**, **4**, **5**, and **2**, respectively. The mixture was recrystallized from benzene (50 mL, rt in the dark) to give hexabromide **3** in 1.67 g (86%) yield.

**Entry 4.** To dibromide **2** (1 g, 2.95 mmol) in benzene (60 mL) was added silica gel (2.23 g) and bromine (1.87 g, 11.70 mmol) in benzene (10 mL). The reaction mixture was stirred for 4 d at room temperature. After completion of the reaction, the resulting material was extracted with saturated Na_2_S_2_O_3_ solution and combined organic layers were washed with water, and dried over anhyd MgSO_4_. After evaporation of the solvent, the residue consisted of compounds **3, 4, 5**, and **2** in the ratio of 80:6:4:10, respectively, as assigned by ^1^H NMR. Recrystallization of the residue from CHCl_3_ (40 mL) gave hexabromide **3** (1.43 g, 74%).

**Entry 5.** To the solution of dibromide **2** (1 g, 2.98 mmol) in CCl_4_ (30 mL) in a cylindrical flask (35 mL) was added bromine (1.2 g, 7.44 mmol). The stirred solution was irradiated with a sun lamp (projector lamp, 150 W) for 1 h at room temperature. After decantation of the precipitated product (1.57 g) during the reaction, the mother liquor was allowed to crystallize in a freezer. After the reaction, the investigation of the reaction mixture by ^1^H NMR indicated that compounds **3, 4**, and **5** were in the ratio of 98:1:1, respectively. Precipitated materials (hexabromide **3**) were combined and recrystallized from CH_2_Cl_2_ (yield: 1.84 g, 95%). Hexabromide **3** is sensitive to daylight and slowly aromatizes to 9,10-dibromoanthracene (**2**) while the product is stable in the dark in the freezer.

### 2. Irradiation of hexabromide **4** in daylight

A solution of hexabromide **4** (50 mg, mmol) in CDCl_3_ was placed in an NMR tube (0.5 mL). The solution was allowed to stand at room temperature in daylight for 1 d. ^1^H NMR analysis indicated the formation of a mixture consisting of **4**, **5**, **3**, and **2** in a ratio of 51:41:5:3. Product ratios were 43:42:6:9 after 3 d and 43:41:7:9 after 22 d for the same compounds (**4**, **5**, **3**, and **2**, respectively) as assigned by ^1^H NMR spectra. When a solution of hexabromide **3** was left to stand in the dark for 3 d in the same conditions, no conversion occurred.

### 3. Irradiation of hexabromide **4** with a projector lamp

A solution of hexabromide **4** (50 mg) in CDCl_3_ was placed in an NMR tube (0.5 mL). When hexabromide **4** was subjected to direct irradiation with a projector lamp (150 W), the equilibrium mixture formed in a short time (15 min). Product distributions are shown in Table 2.

### 4. Base-induced elimination of hexabromide **3**

**NaOCH**_3_. NaOCH_3_ (0.2 g, 3.8 mmol) in dry and freshly distilled THF (20 mL) was added dropwise to a solution of hexabromide **3** (1 g, 1.52 mmol) in dry and freshly distilled THF (20 mL). The mixture was stirred at room temperature overnight under argon atmosphere. After the reaction was complete (TLC control), the reaction material was diluted with diethyl ether (50 mL), washed with H_2_O (3 × 25 mL) and dried over CaCl_2_. After removal of the solvent, the residue was filtered through a short silica gel column (10 g, eluting with hexane). Then the relative percentages of the products were determined by ^1^H NMR as 40:57:3 for tetrabromide **11** [24], tribromide **12**, and dibromide **2**, respectively. Fractional and chromatographic attempts to separate the mixture failed.

**DBU.** To a stirred solution of hexabromide **3** (1.4 g, 2.12 mmol) in dry and freshly distilled THF (80 mL) was added 1,8-diazabicyclo[5.4.0]undec-7-ene (DBU, 0.81 g, 5.33 mmol) dropwise in THF (15 mL) over 15 min. The reaction mixture was stirred at room temperature for 5 h. After completion of the reaction, the reaction mixture was diluted with diethyl ether (50 mL). The organic layer was washed with H_2_O (3 × 40 mL) and dried over CaCl_2_. After removal of the solvent, the residue (0.84 g) was filtered through a short silica gel column (10 g, hexane). Fractional and chromatographic attempts to separate the residue failed. Relative percentages of the products were 22:66:3 for tetrabromide **11**, tribromide **12**, and dibromide **2**, respectively (from ^1^H NMR).

**NaOH.** To the solution of hexabromide **3** (1.0 g, 1.52 mmol) in THF (70 mL) at 0 °C was added NaOH (1.38 g, 34.5 mmol) in THF (20 mL THF + 5 mL H_2_O) over 10 min. The reaction mixture was stirred at 0 °C in the dark for 3 h. After the reaction was complete, the reaction material was diluted with CH_2_Cl_2_ (60 mL) and washed with H_2_O (40 × 4 mL). After drying with CaCl_2_ and removing the solvent, dibromide **2** was obtained in 95% (0.64 g) yield.

### 5. Synthesis of 2,9,10-tribromoanthracene (**12**)

Hexabromide **3** (2.5 g, 3.8 mmol) was dissolved in dry and freshly distilled pyridine (50 mL) at 0 °C. The reaction mixture was stirred at room temperature overnight. After completion of the reaction (TLC control), pyridine was removed *in vacuo*. The residue was diluted with ether (50 mL) and washed with HCl solution (1.56 M, 100 mL). After removal of the solvent, the precipitated material (tribromide **12**) was filtered through a short silica gel column (10 g, eluting hexane) and recrystallized from chloroform/hexane (1.19 g, 75%), yellow needles, m.p. 169 °C . ^1^H NMR (400 MHz, CDCl_3_) δ 8.75 (s, 1H, H_1_), 8.55 (m, 2H, H_5_ and H_8_), 8.3 (d, 1H, *J*_34_ = 7.6 Hz, H_3_), 7.57 (d, 1H, H_4_), 7.57 (m, 2H, H_6_ and H_7_); ^13^C NMR (100 MHz, CDCl_3_) δ 133.1, 133.0, 132.8, 132.1, 131.9, 130.3, 130.2, 130.0, 129.7, 129.4, 125.7, 125.5, 124.4, 124.1; IR (KBr) ν_max_ 3025, 1616, 1602, 1438, 1421, 1292, 1072, 1065, 862, 802, 748; MS (CI) *m/z* 411.77/413.76/414.77/415.76/417.76 (M^+^), 331.88/333.88/334.88/335.88/336.88 (M^+^–Br), 254.00/255.00/256.00/257.00 (M^+^–2Br), 173.08/174.08/175.08/176.08 (M^+^–3Br), 149.08/128.05/127.05/110.06; Anal. calcd for C_14_H_7_Br_3_ (414.9): C, 40.53; H, 1.70. Found: C, 40.65; H, 1.63.

### 6. Synthesis of 2,9,10-trimethoxyanthracene (**13**)

Freshly cut sodium (1.17 g, 51 mmol) was added to dry methanol (60 mL) under nitrogen gas. When dissolution was completed, the warm solution was diluted with dry DMF (60 mL), which was followed by the addition of vacuum-dried copper(I) iodide (1.0 g, 5.2 mmol). After dissolution, tribromide **12** (1.40 g, 3.40 mmol) in dry DMF (70 mL) was added. The reaction mixture was stirred magnetically under argon gas atmosphere at reflux (ca. 110 °C) for 44 h. Reaction progress was monitored by TLC. After cooling to room temperature, diethyl ether (80 ml) and H_2_O (70 mL) were added to the reaction mixture. The organic layer was separated, washed with H_2_O (3 × 60 mL), dried over CaCl_2_, and concentrated at reduced pressure. The crude product was passed through a short column packed with aluminum oxide (10 g), (*R_f_* = 0.41, hexane/EtOAc, 9:1). Recrystallization from chloroform/hexane in the refrigerator yielded 2,9,10-trimethoxyanthracene (**13**, 0.63 g, 70%) as colorless needles, m.p. 139–140 °C. ^1^H NMR (400 MHz, CDCl_3_) δ 8.38 (m, 3H, H_4_, H_5_, H_8_), 7.59 (m, 2H, H_6_, H_7_), 7.54 (brs, 1H, H_1_), 7.28 (dd, 1H, H_3_, *J*_34_ = 9.37 Hz, *J*_31_ = 1.47 Hz), 4.25 (s, 3H, OMe), 4.24 (s, 3H, OMe), 4.12 (s, 3H, OMe); ^13^C NMR (50 MHz, CDCl_3_) δ 157.8, 149.2, 147.0, 126.2, 125.9, 125.7, 124.8, 124.5, 123.8, 123.1, 122.5, 121.8, 120.8, 98.3, 63.3, 62.3, 55.4; MS (CI) *m/z* 268 (M^+^), 253 (M^+^–CH_3_), 238 (M^+^–2CH_3_), 223 (M^+^–3CH_3_), 209, 195, 181, 167, 152, 139, 134, 119, 111; IR (KBr) ν_max_ 3000, 2950, 2837, 1631, 1363, 1274, 1230, 1063, 1028, 964, 846, 825, 771, 721, 551. Anal. calcd for C_17_H_16_O_3_ (268.3): C, 76.10; H, 6.01; O: 17.89. Found C: 75.96; H, 6.10; O, 17.95.

### 7. Preparation of 2,9,10-tricyanoanthracene (**14**)

2,9,10-Tribromoanthracene (**12**, 1 g, 2.4 mmol) dissolved in freshly distilled DMF (80 mL) was mixed with CuCN (1.3 g, 14.5 mmol). The reaction mixture was stirred magnetically at reflux (ca. 150 °C) under argon for 7 h. The hot resulting brown mixture was poured (residues are conveniently transferred with hot DMF) into a solution of hydrated ferric chloride (6 g) and concentrated HCl (2.5 mL) in water (15 mL). After the reaction mixture had been maintained at 70 °C for 20 min to decompose the complex, the layers were separated (light reflected off the separatory funnel helps, or differences in fluidity of the two layers may be discerned as the liquid leaves the separatory funnel). The hot aqueous layer was extracted with hot toluene (4 × 80 mL) and the combined extracts were washed with dilute hydrochloric acid (100 mL, 1:1), aqueous sodium hydroxide (100 mL, 10%), and water (100 mL), in that order. The organic layer was dried over Na_2_SO_4_. The crude product was filtered through a short silica gel (20 g) column, and recrystallization from toluene, allowing the sample to stand in the refrigerator (ca. 5 °C), afforded 0.456 g (75%) of pure 2,9,10-tricyanoanthracene (**14**). Yellow needles, m.p. 256–257 °C.^ 1^H NMR (400 MHz, CDCl_3_) δ 8.95 (d, *J*_13_ = 0.4 Hz, 1H, H_1_), 8.65 (dd, *J*_34_ = 9.4, H_4_, 1H), 8.61–8.58 (dd, 2H, H_5_ and H_8_), 7.99–7.94 3H, H_3_, H_7_ and H_6_); ^13^C NMR (50 MHz, CDCl_3_) δ 133.6, 132.9, 132.6, 132.1, 131.5, 131.1, 130.4, 129.2, 127.8, 126.5, 126.4, 117.4, 115.0, 114.9, 113.7, 112.8, 112.3; MS (APCI) *m/z* 253 (M^+^), 268 (M^+^+Na+NH_4_–CN), 243 (M^+^–H+Na+NH_4_–2CN), 215; IR (KBr) ν_max_ 3063, 2926, 2218, 1623, 1542, 1512, 1449, 1433, 1382, 1355, 1284, 1267, 1177, 977, 911, 824, 760, 660, 620, 562, 504, 489, 451, 429.

## Supporting Information

File 1 NMR spectra for the new compounds
