# Supplementary material for: Highly brominated anthracenes as precursors for the convenient synthesis of 2,9,10-trisubstituted anthracene derivatives
Source: Beilstein J Org Chem. 2008 Dec 10;4:50. doi: 10.3762/bjoc.4.50 (PMC2633663; doi:10.3762/bjoc.4.50)
Supplement: File 1 — NMR spectra for the new compounds [file Beilstein_J_Org_Chem-04-50-s001.doc]

**Supporting Information (NMR Spectra)**

Highly brominated anthracenes as precursors for the convenient synthesis of 2,9,10-trisubstituted anthracene derivatives

Osman Cakmak*^,1^, Leyla Aydogan^1^, Kiymet Berkil^1^, Ilhami Gulcin^2^ and Orhan Buyukgungor^3^

Address: ^1^Gaziosmanpasa University, Faculty of Art and Science, Department of Chemistry, TR-60240, Tokat, Turkey, ^2^Ataturk University, Faculty of Art and Science, Department of Chemistry, TR-25240, Erzurum, Turkey and ^3^Ondokuzmayıs University, Faculty of Art and Science, Department of Physics, TR-55060, Samsun, Turkey

**
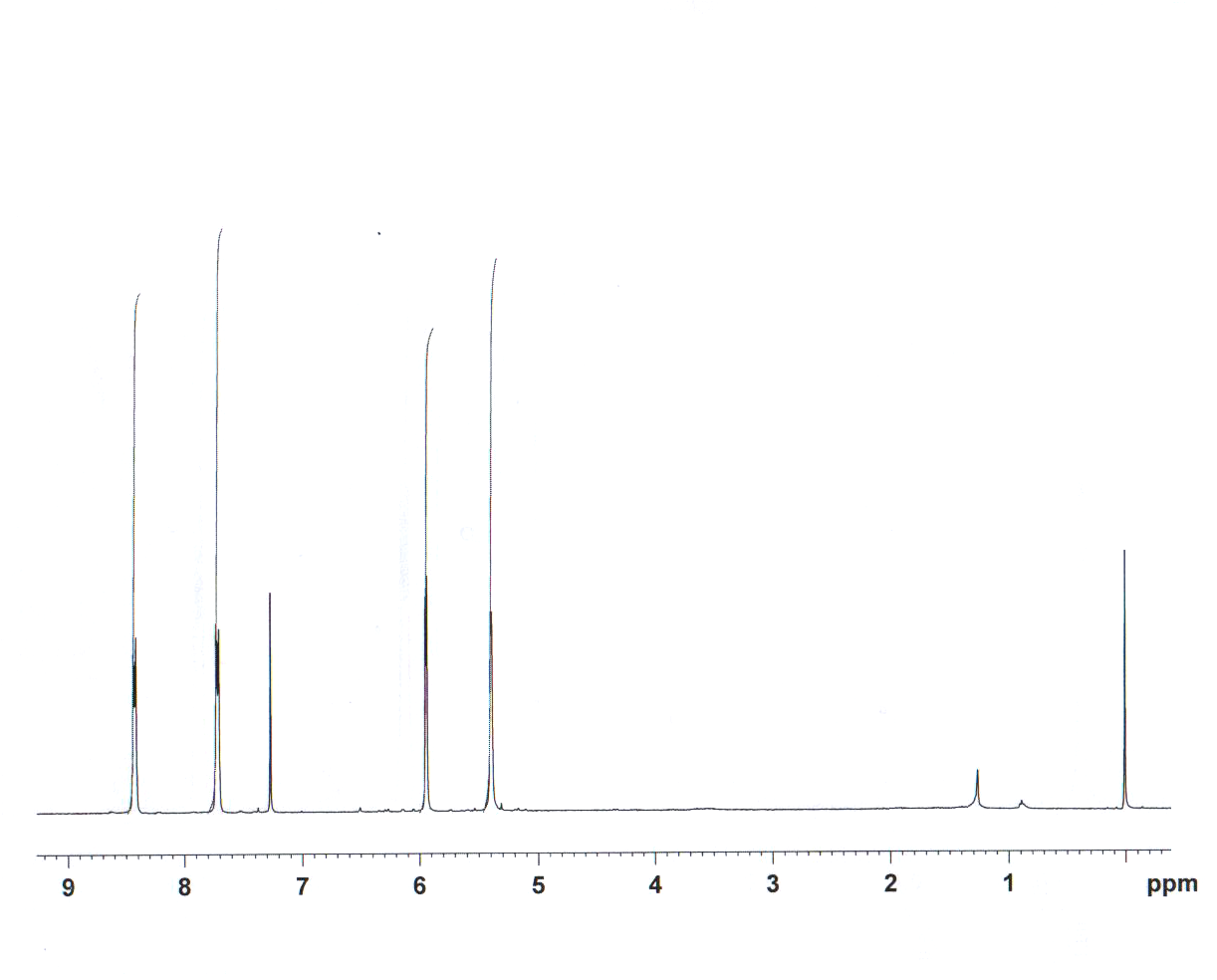
**

**3**


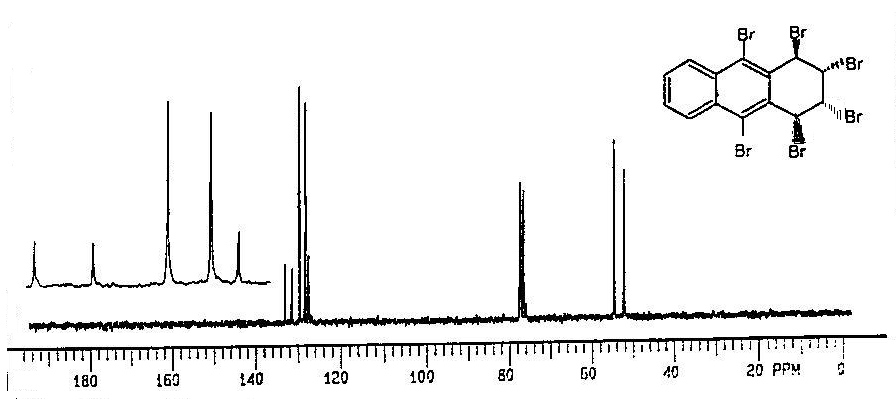


**3**

**
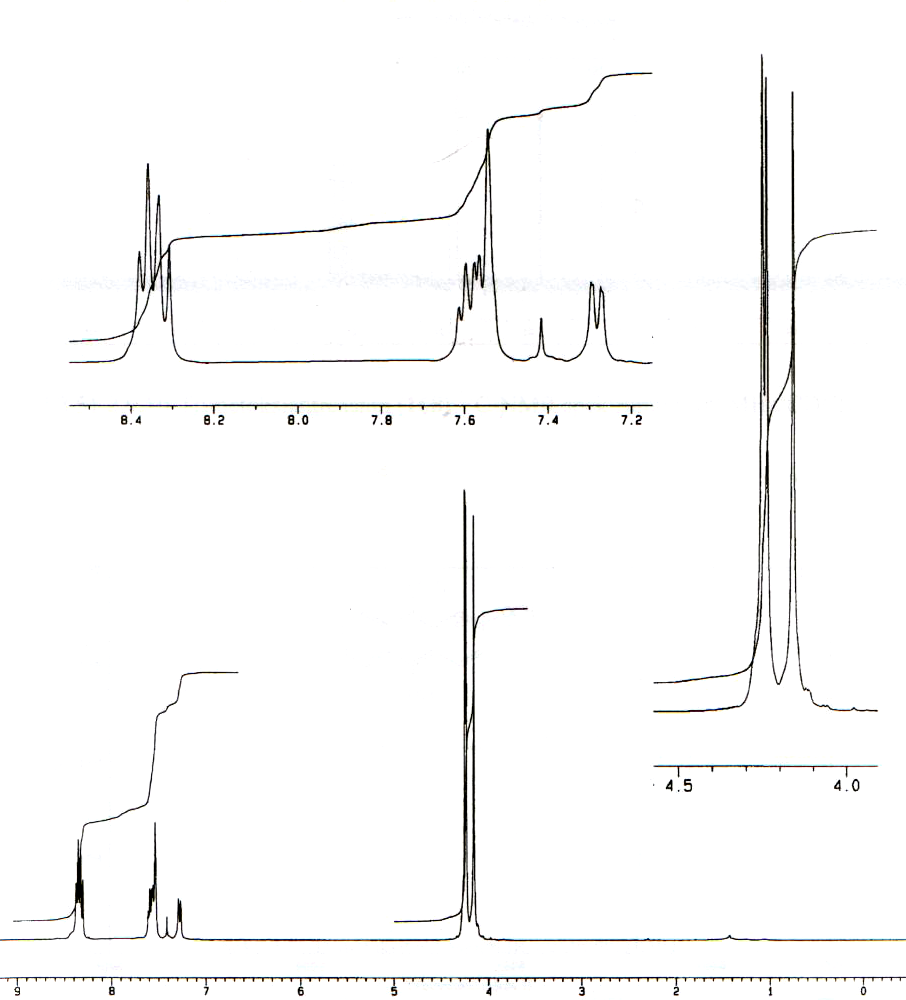
**

**13**

**
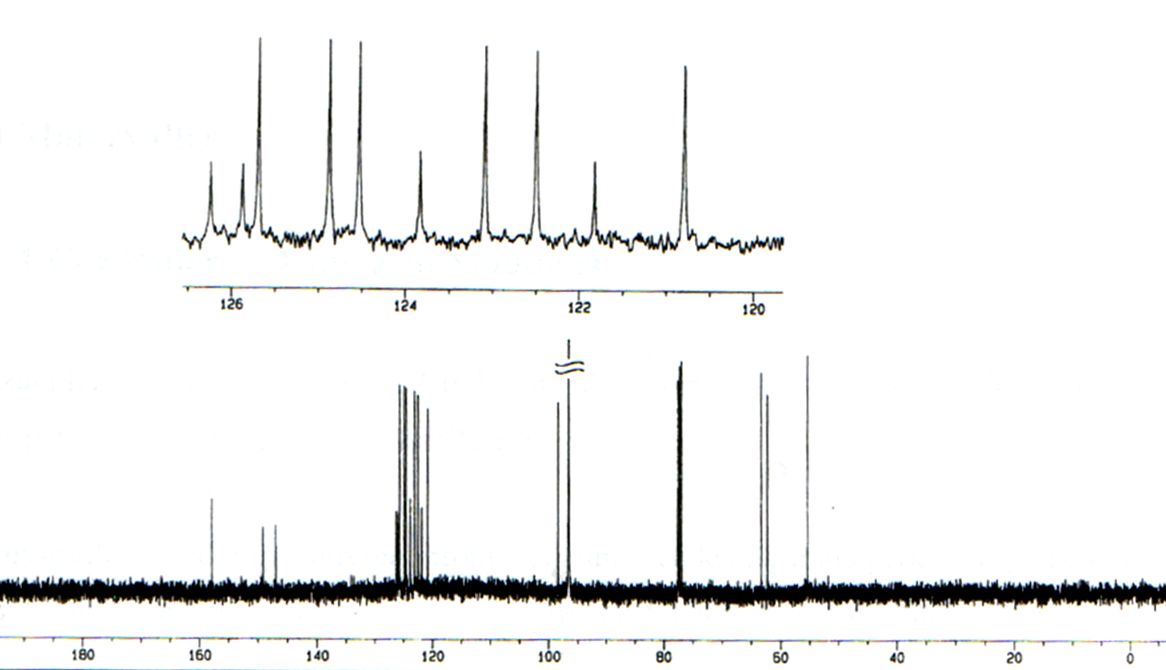
**

**13**


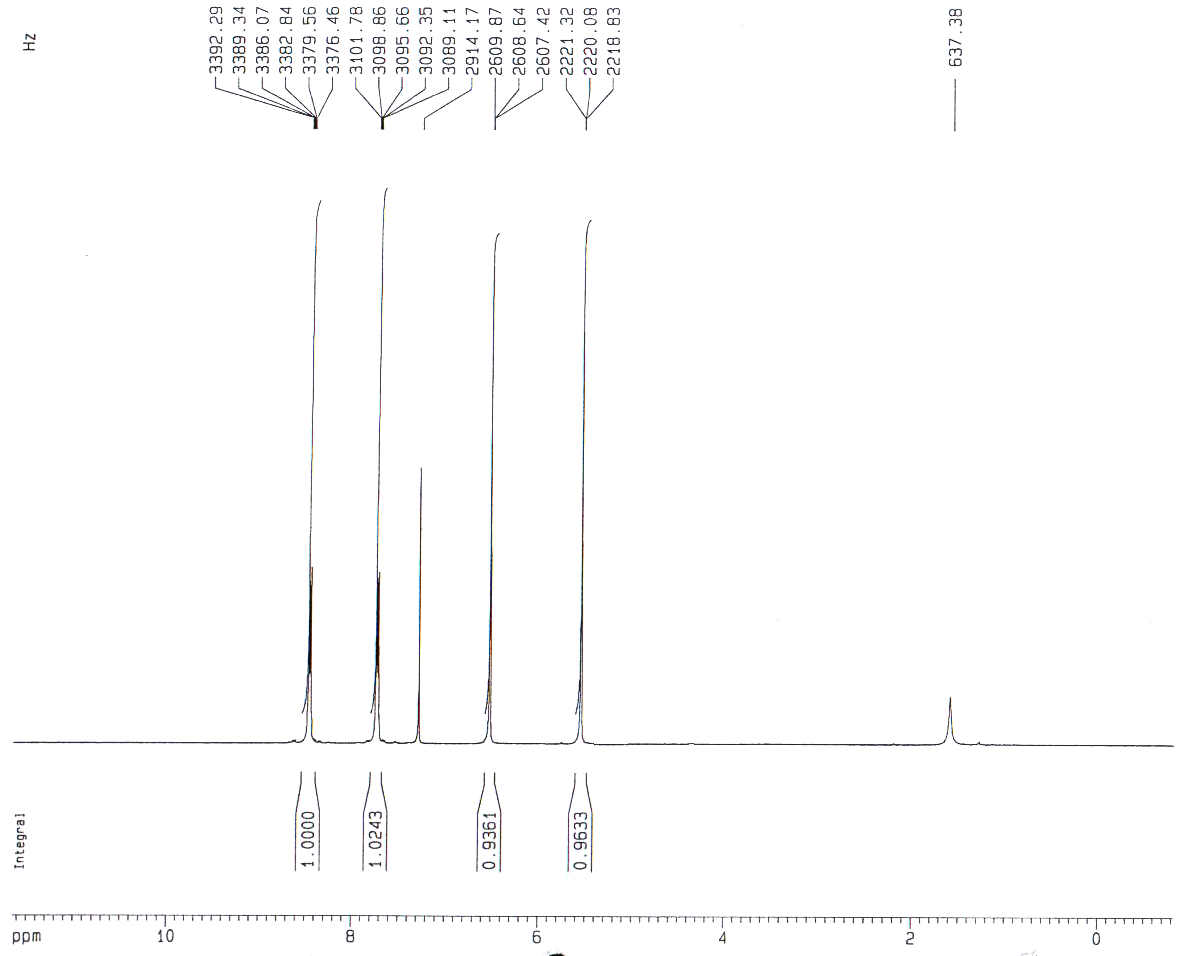


**5**

**5**


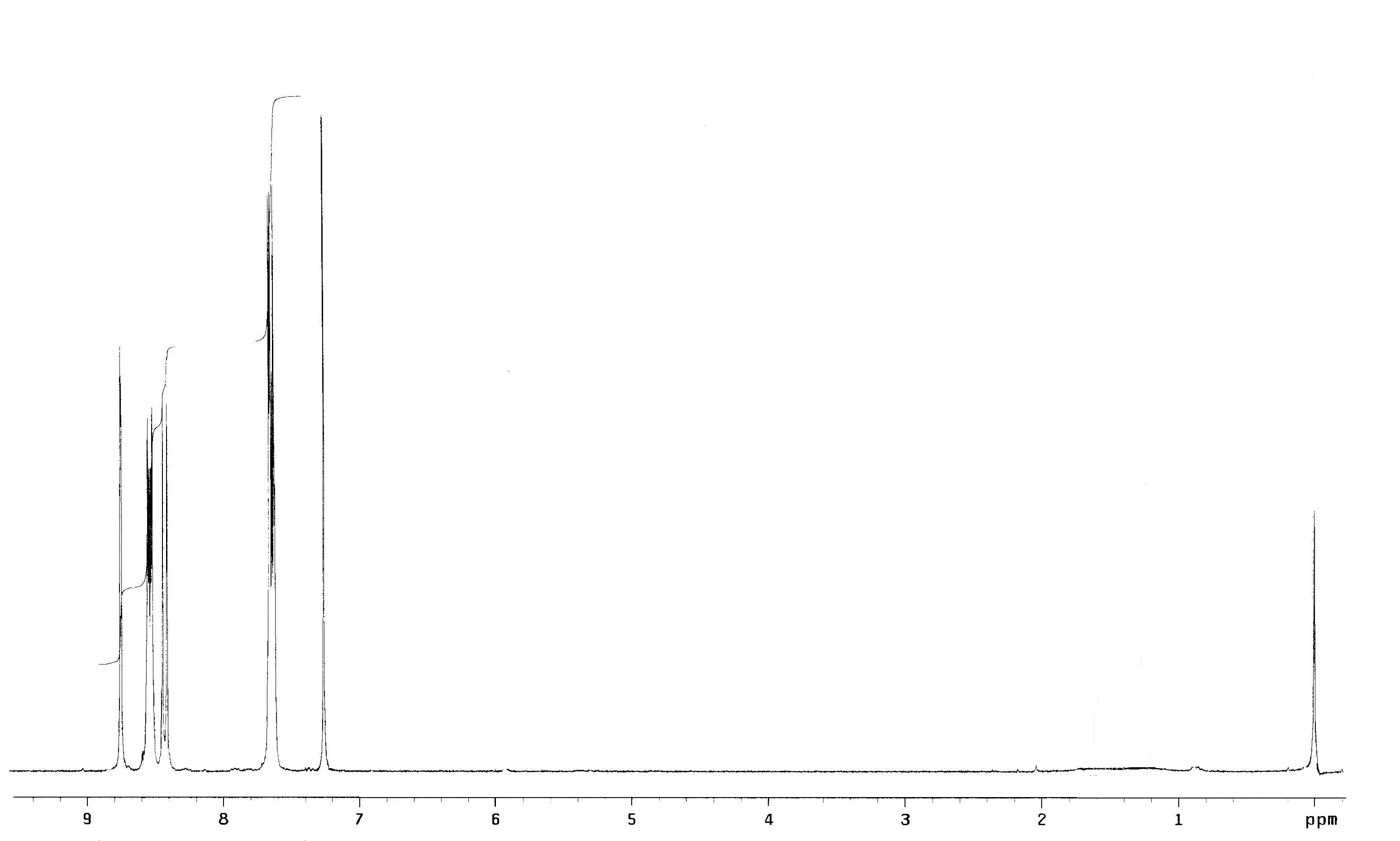


**12**


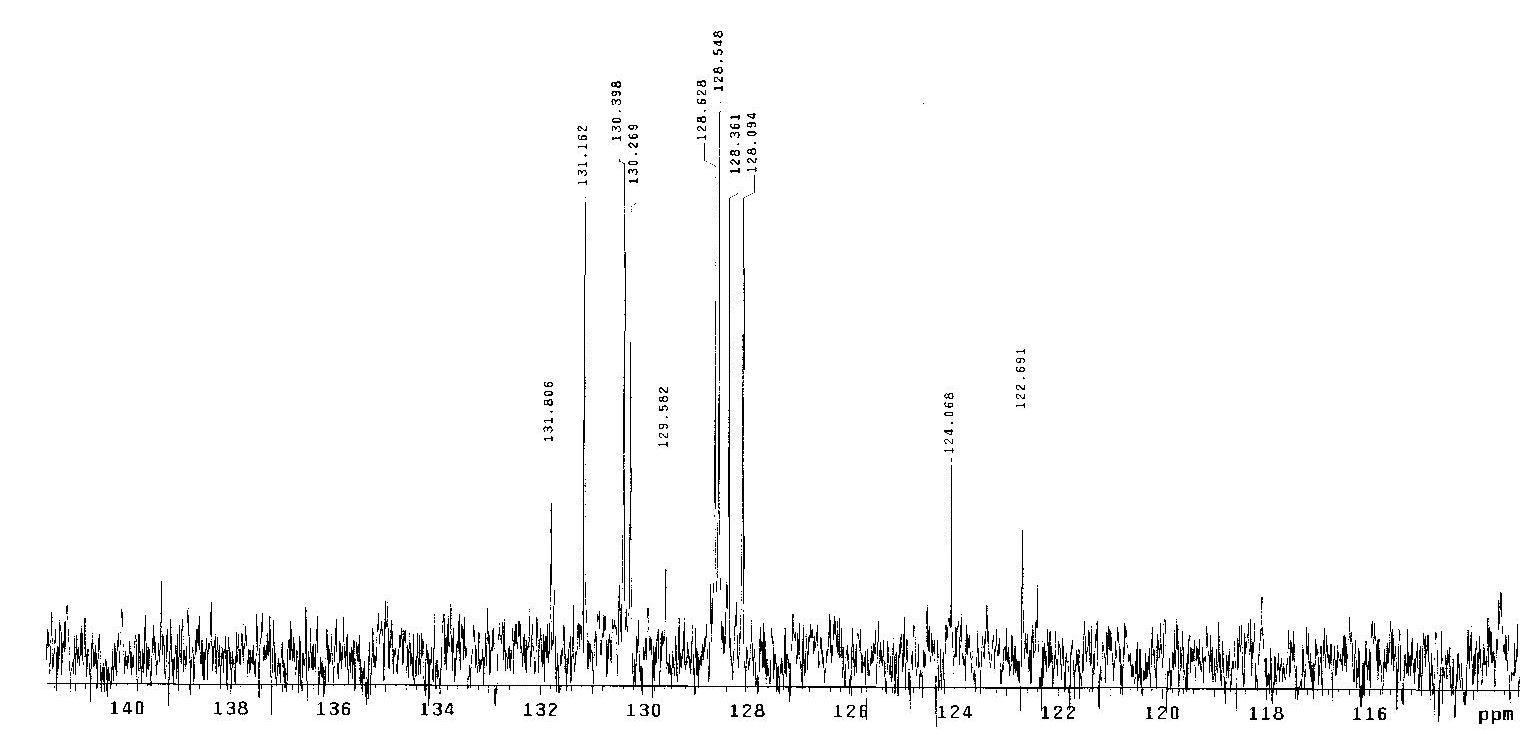


**12**

**
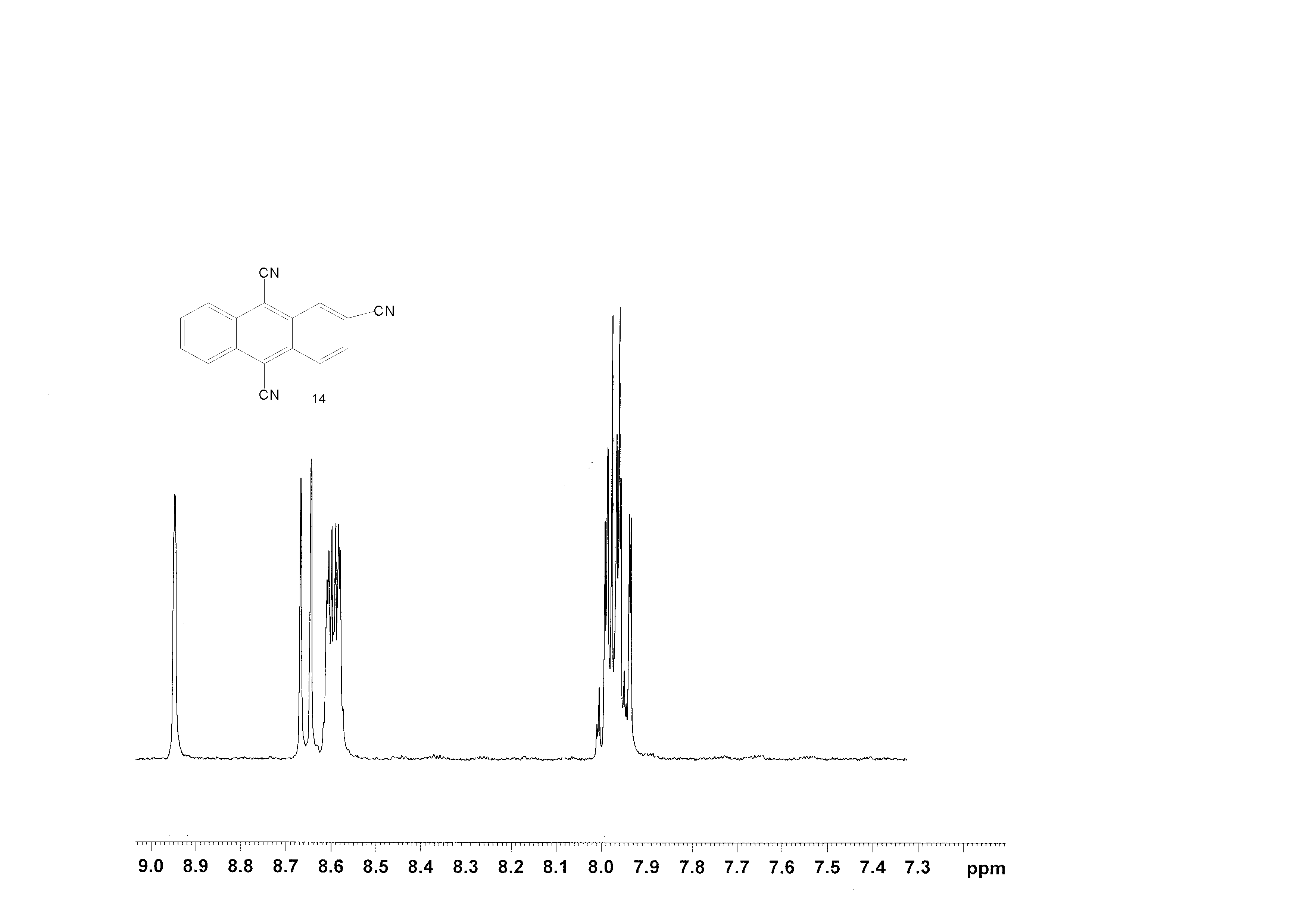
**

**
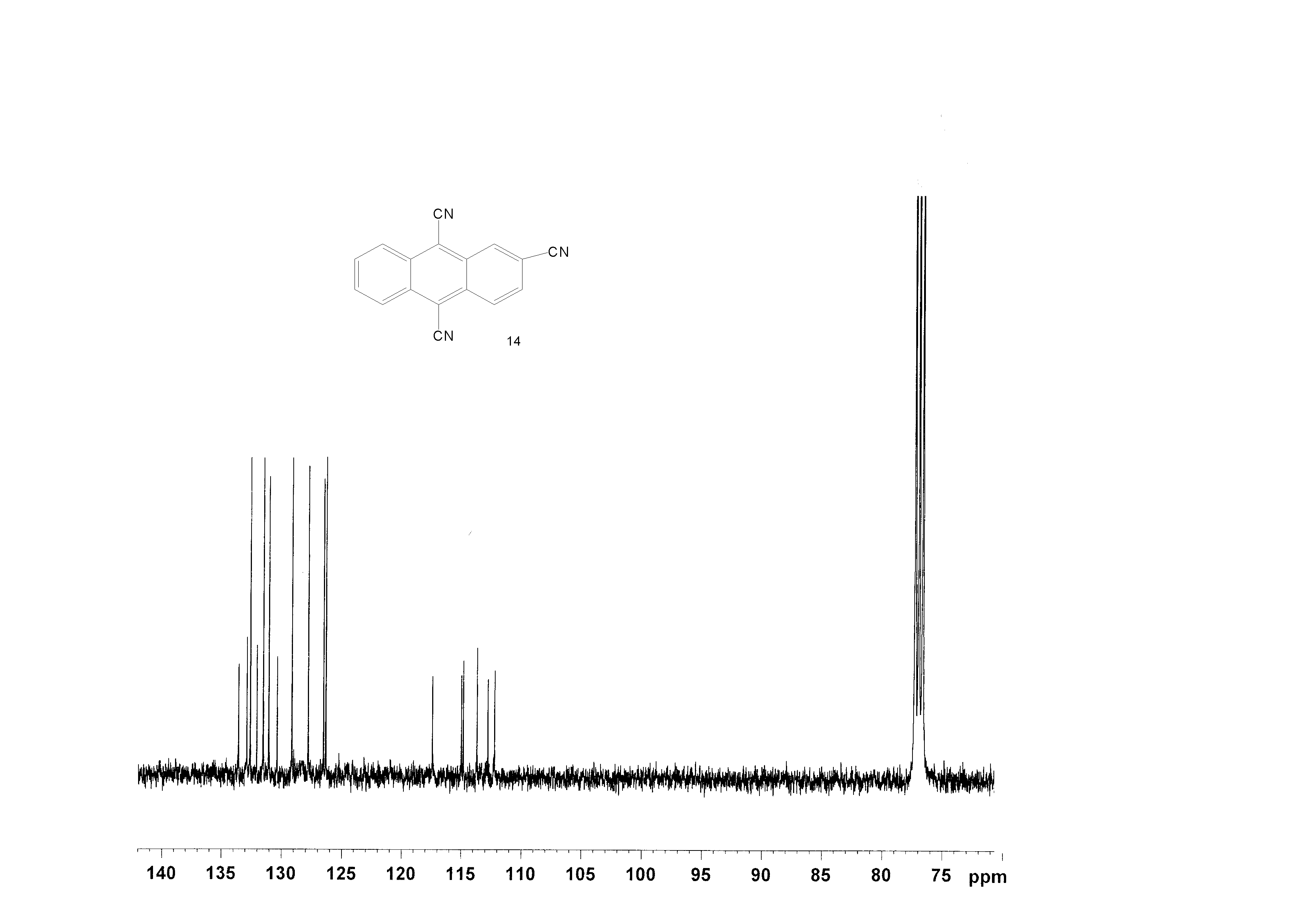
**
